# Characterization of Three Calcium-Dependent Protein Kinases of *Cryptosporidium parvum*

**DOI:** 10.3389/fmicb.2020.622203

**Published:** 2021-01-12

**Authors:** Qiang Zhang, Qian Shao, Yaqiong Guo, Na Li, Yu Li, Jiayuan Su, Rui Xu, Ziding Zhang, Lihua Xiao, Yaoyu Feng

**Affiliations:** ^1^State Key Laboratory of Bioreactor Engineering, School of Resource and Environmental, East China University of Science and Technology, Shanghai, China; ^2^Center for Emerging and Zoonotic Diseases, College of Veterinary Medicine, South China Agricultural University, Guangzhou, China; ^3^State Key Laboratory of Agrobiotechnology, College of Biological Sciences, China Agricultural University, Beijing, China

**Keywords:** *Cryptosporidium parvum*, calcium-dependent protein kinase, invasion, intracellular development, inhibitor

## Abstract

In *Cryptosporidium* spp., calcium-dependent protein kinases (CDPKs) are considered promising targets for the development of pharmaceutical interventions. Whole-genome sequencing has revealed the presence of 11 CDPKs in *Cryptosporidium parvum* (CpCDPKs). In this study, we expressed recombinant CpCDPK4, CpCDPK5, and CpCDPK6 in *Escherichia coli*. The biological characteristics and functions of these CpCDPKs were examined by using quantitative reverse transcription PCR (qRT-PCR), immunofluorescence microscopy, and an *in vitro* neutralization assay. The expression of the CpCDPK4 gene peaked at 12 h post-infection, the CpCDPK5 gene peaked at 12 and 48 h, and the CpCDPK6 gene peaked at 2–6 h. CpCDPK4 protein was located in the anterior and mid-anterior regions of sporozoites, and CpCDPK5 protein was located over the entire sporozoites, while CpCDPK6 protein was expressed in a spotty pattern. Immune sera of CpCDPK4 and CpCDPK6 exhibited significant inhibitory effects on host cell invasion, while the immune sera of CpCDPK5 had no effects. These differences in protein localization, gene expressions, and neutralizing capacities indicated that the CpCDPK proteins may have different roles during the lifecycle of *Cryptosporidium* spp.

## Introduction

*Cryptosporidium* spp. are apicomplexan parasites causing enterocolitis, vomiting, and watery diarrhea in humans and animals worldwide (Kotloff et al., 2013; Checkley et al., 2015). The infection is self-limited in immunocompetent hosts but can persist for a significant duration in immunocompromised hosts (Wang et al., 2018; Bones et al., 2019). To date, nitazoxanide is the only drug approved by the United States Food and Drug Administration against cryptosporidiosis, but it is ineffective for AIDS patients and malnourished children (Abubakar et al., 2007, 2010; Amadi et al., 2009). The lack of effective treatment is partially attributed to the poor understanding of the invasion and intracellular development of *Cryptosporidium* spp. (Bhalchandra et al., 2018).

Previous studies have shown that calcium ions are involved in many critical events during the lifecycle of apicomplexan parasites, such as protein secretion, gliding motility, host invasion, and egress (Billker et al., 2009). In these pathogens, the regulation of calcium ions is in response to the activity of calcium-dependent protein kinases (CDPKs), which are found in plants, ciliates, and apicomplexan protozoa, but not in fungi and vertebrates. Because of this, CDPKs are considered ideal targets for the development of treatments against cryptosporidiosis (Harper and Alice, 2005; Hui et al., 2015). To date, whole-genome sequencing and transcriptomic analysis have revealed the presence of 11 CDPK proteins in *Cryptosporidium parvum* (Cp; Lippuner et al., 2018).

Most previous studies of CDPKs in *C. parvum* (CpCDPKs) had focused on CpCDPK1, which has been shown to play an important role in the invasion process (Castellanos-Gonzalez et al., 2016; Kuhlenschmidt et al., 2016). Recently, CpCDPK3 was reported to be involved in the intracellular development of *C. parvum*. In comparison, the functions of other CpCDPKs have not been examined.

We have expressed in the study the recombinant proteins of CpCDPK4 encoded by the cgd7_40 gene, CpCDPK4 encoded by the cgd2_1300 gene, and CpCDPK4 encoded by the cgd4_3330 gene and examined their potential roles in the lifecycle of *C. parvum* through immunofluorescence microscopy, quantitative RT-PCR analysis, and *in vitro* neutralization assays. The anti-cryptosporidial effects of some small molecules selected by the molecular docking of the CpCDPKs were assessed.

## Materials and Methods

### Parasite and Cell Culture

Oocysts of the *C. parvum* IOWA strain were purchased from Waterborne, Inc. (New Orleans, United States) and stored at 4°C in phosphate-buffered saline (PBS) with 200 U/ml penicillin, 200 μg/ml streptomycin, and 0.5 μg/ml amphotericin B. They were used within 3 months. Prior to use, oocysts were treated on ice with chilled 0.5% sodium hypochlorite for 10 min and washed three times with PBS by centrifugation at 13,200 × *g* for 3 min. For excystation and harvesting sporozoites, the sodium hypochlorite-treated oocysts were incubated with D-Hanks buffer containing 0.25% trypsin and 0.75% sodium taurocholate at 37°C for 1 h. The released sporozoites were collected and washed three times with PBS by centrifugation at 13200 × *g* for 2 min.

Human colon adenocarcinoma cells (HCT-8 cells) were purchased from the Chinese Academy of Sciences. The cells were cultured at 37°C and 5% CO_2_ in RPMI 1640 medium supplemented with 10% fetal bovine serum (FBS), 100 U/ml penicillin, and 100 μg/ml streptomycin.

### Domain Prediction and Phylogenetic Analyses of CpCDPKs

The CpCDPK4 (cgd7_40), CpCDPK5 (cgd2_1300), and CpCDPK6 (cgd4_3330) genes were identified from the whole genome sequences of *C. parvum* IOWA in the CryptoDB database.^1^ The protein kinase domain, EF-hand domain, and active site of these CDPKs were predicted by sequence analysis using HMMER (Potter et al., 2018).^2^ The phylogenetic relationship among CDPK proteins of *C. parvum*, *Toxoplasma gondii* (Tg), and *Plasmodium falciparum* (Pf) was assessed by using the maximum likelihood method implemented in MEGA-X 10.0.5, based on the substitution rates calculated with the JTT matrix-based model. Bootstrap values were obtained by running 1,000 replicates.

### Cloning, Expression, and Purification of Recombinant CpCDPKs and Preparation of Polyclonal Antibodies

Full-length *Cp*CDPK genes were amplified using PCR from genomic DNA extracted from oocysts of the *C. parvum* IOWA strain. For cgd7_40, the primers used included 5'-CGGAATTCATGGAAAAGAACCGA-3' (with *Eco*R I restriction enzyme site underlined) and 5'-CCGCTCGAGACTGTCACATAACAG-3' (with *Xho* I restriction enzyme site underlined). For cgd2_1300, the primers used were 5'-CGCGGATCCATGTTAAATATAGAACAAAATGC-3' (with *Bam*H I restriction enzyme site underlined) and 5'-CCGCTCGAGATTATTCAGCTTCTTAAAAATG-3' (with *Xho* I restriction enzyme site underlined). For cgd4_3330, the primers used were 5'-CGCGGATCCATGAGTAGTGAATATA-3' (with *Bam*H I restriction enzyme site underlined) and 5'-CCGCTCGAGGTTAATCATGTAATCC-3' (with *Xho* I restriction enzyme site underlined). The PCR products were purified by using the E.Z.N.A® Cycle-Pure Kit (Omega Bio-Tek, Norcross, United States), double-digested with *Eco*RI/*Bam*H I and *Xho*I restriction enzymes (New England Biolabs, Ipswich, United States), and ligated into the pET-28a-c(+) vector (Novagen, Madison, United States). The ligation products were used to transform *Escherichia coli* DH5α competent cells. Positive colonies were identified using PCR and verified by DNA sequencing. The recombinant vectors were extracted by using E.Z.N.A® Plasmid Mini Kit (Omega Bio-Tek). The recombinant *Cp*CDPKs-pET-28a-c(+) plasmids were transformed into *E. coli* BL21(DE3) competent cells, which were cultured in LB medium supplemented with 100 μg/ml kanamycin for protein expression. The expression was induced by adding 0.5 mM isopropylthio-β-galactoside (IPTG) to the cultures maintained at 25°C for 8 h. The expression level of recombinant CpCDPK proteins was assessed by using sodium dodecyl sulfate polyacrylamide gel electrophoresis (SDS-PAGE) and Western blot analysis with anti-His-tag antibodies.

For protein purification, cultured BL21(DE3) cells were collected by centrifugation and lysed by sonication on ice. The lysate was centrifuged to separate the supernatant and sediment. For CpCDPK6, the supernatant was filtered through a 0.45-μm polyvinylidene fluoride (PVDF) membrane filter (Millipore, Billerica, MA, United States) and loaded onto a column containing Ni-NTA His-bind resins (Novagen) at room temperature. *Cp*CDPK6 was eluted from the resin with 250 mM imidazole buffer. For CpCDPK4 and CpCDPK5, the proteins were purified with gel extraction. Briefly, the inclusion body in the sediment was dissolved with PBS containing 8 M urea and centrifuged to remove the undissolved pellet. The dissolved protein solution was filtered through a 0.45-μm PVDF membrane filter and used for SDS-PAGE to separate target proteins. The bands of target proteins were cutoff from the gel and dialyzed in dialysis bags containing SDS-PAGE buffer. The purified proteins were examined using SDS-PAGE and Western blot. The Matrix-Assisted Laser Desorption/Ionization Time of Flight Mass Spectrometry (MALDI-TOF/MS; Applied Protein Technology, Shanghai, China) was used to analyze the bands in SDS-PAGE for the verification of the protein identity.

Polyclonal antibodies against *Cp*CDPKs were generated through immunizations of specific pathogen-free rabbits with Freund’s complete and incomplete adjuvants by GL Biochem Ltd (Shanghai, China). After the final immunization, the post-immune serum was collected from the rabbits. Polyclonal antibodies were purified by affinity chromatography with purified recombinant proteins. The titer and specificity of the antibodies were assessed using ELISA and Western blot, respectively.

### Assessment of CpCDPK Gene Expression in Developmental Stages

The expression of the *Cp*CDPK genes in intracellular stages of *C. parvum* was assessed by using quantitative reverse transcription PCR (qRT-PCR) as described (Mauzy et al., 2012). HCT-8 cells were seeded into 12-well plates and cultured until 60% confluence. Prior to infection, the concentration of FBS was reduced to 2% in the culture medium. Sodium hypochlorite-treated oocysts were inoculated onto cells (5 × 10^5^ oocysts/well) and incubated with cells at 37°C for 2 h. After incubation, the cells were washed with PBS three times and cultured in fresh medium with 2% FBS. Total RNA was isolated from the *C. parvum*-infected cells at 2, 6, 12, 24, 36, 48, and 72 h post-infection using the RNeasy Mini kit (QIAGEN, Hilden, Germany). For cDNA Synthesis, 1 μg RNA was reverse-transcribed using the RevertAid First Strand Kit (Thermo Fisher Scientific, Waltham, United States). qPCR analysis of the cDNA was conducted in a 20 μl mixture which contained 10 μl 2×SYBR Green Real-Time PCR Master Mix (Toyobo, Osaka, Japan), 0.5 mM primers, and 1 μl cDNA in a Light Cycler 480 Instrument II (Roche, Basel, Switzerland). The *Cp*CDPK genes were amplified by using the following primers for cgd7_40: 5'-TGCTTCGAGAATCCAAACTACA-3' and 5'-CAATTCCAGCGATAGGACTCA-3'; cgd2_1300: 5'-TCGGACTCTTCTCCAAACTCTC-3' and 5'-CTGCGCTCCATAAATCACATAA-3'; and cgd4_3330: 5'-TATGGTGTGGATGACCAGGA-3' and 5'-ATTGGAGGGAGCTCCAAGTT-3'. Data from concurrent analysis of the 18S rRNA gene of *C. parvum* were used in data normalization as described (Mauzy et al., 2012). The relative expression level of the *Cp*CDPK genes at different time points was calculated with the 2^-△△C^_T_ method as described (Livak and Schmittgen, 2001). The results were based on the mean values of two technical replicates for each cDNA from three independent biological experiments.

### Assessment of Expression of Native CpCDPK Proteins

Western blot was used in the assessment of native CpCDPK proteins in the crude protein extract of excysted sporozoites (~5 × 10^6^ oocysts/lane). Briefly, excysted sporozoites were resuspended in PBS, mixed with protease inhibitor cocktail (Merck, Darmstadt, Germany) and protein loading buffer, and incubated in 100°C water bath for 5 min. The native proteins were separated by SDS-PAGE and transferred onto PVDF membranes. Purified anti-*Cp*CDPK antibodies (0.4 μg/ml), antiserum (1:4,000 dilution), or pre-immune serum (1:4,000 dilution) were used as primary antibodies. The horseradish peroxidase-conjugated anti-rabbit IgG (Cell Signaling Technology, Whitby, Canada) was used as the secondary antibody at 1:5,000 dilution.

### Assessment of CpCDPKs Expression in Developmental Stages

Immunofluorescence microscopy was used to examine the *Cp*CDPKs expression in the developmental stages of *C. parvum*. For extracellular stages, *C. parvum* oocysts and excysted sporozoites were fixed with methanol on SuperStick Slides (Waterborne) for 20 min. For intracellular stages, *C. parvum*-infected HCT-8 cells were cultured for 24 and 48 h and fixed with methanol. The two time points were chosen to allow the detection of diverse lifecycle stages due to asynchronized development of the parasites in culture. After fixation, oocysts, sporozoites, and cultured cells were permeabilized with 0.5% Triton X-100 in PBS for 15 min, blocked with 5% bovine serum albumin (BSA) in PBS for 1 h, and incubated with anti-*Cp*CDPK antibodies (0.4 μg/ml) for 1 h. The 1:400 diluted Alexa Fluor® 594-conjugated Goat Anti-rabbit IgG (Cell Signaling Technology, Whitby, United States) was used as the secondary antibody. After incubation, the cell nuclei were counterstained with the 4',6-diamidino-2-phenylindole (DAPI) for 5 min. Three PBS washes were performed after each treatment. The slides were examined under an Olympus BX53 fluorescence microscope (Olympus, Tokyo, Japan).

### *In vitro* Neutralization of *Cryptosporidium parvum* Invasion with Anti-CpCDPK Antibody

The involvements of *Cp*CDPKs in the *C. parvum* invasion were examined by using *in vitro* neutralization assays (Kuhlenschmidt et al., 2016). Briefly, excysted sporozoites were incubated in 2% FBS culture medium containing 1:200, 1:500, and 1:1,000 dilutions of post-immune serum or pre-immune serum at 37°C for 15 min. They were inoculated onto HCT-8 cells monolayer cultured on coverslips at 1 × 10^5^ oocysts/coverslip and incubated for 2 h. After incubation, the cultures were washed with PBS three times and allowed to continue for 24 h. The developmental stages of *C. parvum* were stained with Cy3-labeled Sporo-Glo™ antibody (Waterborne) and examined under a BX53 fluorescence microscope. The number of parasites in each field was determined using the Image J software.^3^ For each coverslip, the mean value of 50 random fields under 200× was used to calculate the parasite load. Data from cultures treated with pre-immune serum in corresponding dilutions were used as controls. All experiments were performed in triplicate.

### Inhibition of *Cryptosporidium parvum* Development with Candidate Inhibitors of CpCDPKs

An *in vitro* neutralization assay was used to assess the anti-cryptosporidial effects of small molecule compounds in the ChemDiv database selected by molecular docking of the *Cp*CDPK structure. The parasite loads in cell culture were quantified by using the one-step qRT-PCR (Zhang et al., 2012). Briefly, HCT-8 cells were cultured in 96-well plates until 80% confluence. They were inoculated with sodium hypochlorite-treated oocysts at 1 × 10^5^ oocysts/well in the presence of 10 μM candidate compounds or DMSO in 2% FBS culture medium as done previously (Zhang and Zhu, 2015). After 2-h cultivation at 37°C, the cultures were washed three times with PBS and allowed to continue in a medium containing the candidate compounds or DMSO for 24 h. Total RNA was extracted from the cultures using the RNeasy Mini kit (QIAGEN). The parasite RNA was quantified as described (Cai et al., 2005) using the HiScript II One Step qRT-PCR SYBR Green Kit (Vazyme, Nanjing, China). At least two technical replicates were used in qRT-PCR analysis of the culture. In a secondary analysis of selective compounds, various concentrations (from 20 nM to 25 μM) of the compounds were used in treating *C. parvum* cell cultures as described above. All experimental infection were performed in triplicate.

## Results

### Function Domains and Phylogeny of CpCDPKs

**Figure 1:**
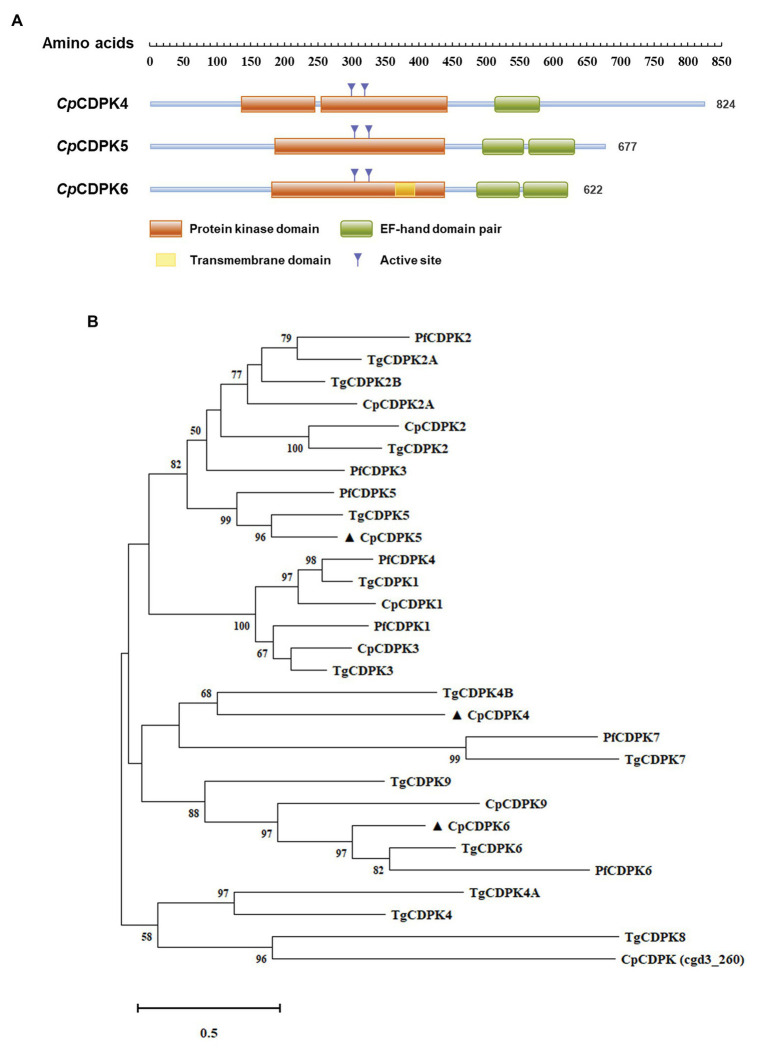
Domain structures and phylogenetic relationship of CpCDPK4, CpCDPK5, and CpCDPK6. **(A)** Predicted domain structures of CpCDPK4, CpCDPK5, and CpCDPK6. In the diagram, functional domains are indicated by boxes. The domain predictions were performed by using the HMMER (https://www.ebi.ac.uk/Tools/hmmer). **(B)** Phylogenetic relationship of the calcium-dependent protein kinases (CDPKs) from *Cryptosporidium parvum* (Cp), *Toxoplasma gondii* (Tg), and *Plasmodium falciparum* (Pf). The tree was generated by using the maximum likelihood method implemented in MEGA-X 10.0.5. Sequences in the present study are labeled with ▲.

Functional domains in the three CpCDPKs were predicted using sequence analysis with the profile hidden Markov Models. The results showed that both CpCDPK5 and CpCDPK6 have one complete protein kinase domain and two EF-hand domain pairs, while CpCDPK4 has only one disconnected protein kinase domain and one EF-hand domain pair (Figure 1A). Moreover, there is a transmembrane domain at 365–383 amino acids in CpCDPK6. The result of phylogenetic analyses showed that CpCDPK5 is most related to TgCDPK5 and PfCDPK5, while CpCDPK6 is most related to TgCDPK6 and PfCDPK6. In contrast, CpCDPK4 is most related to TgCDPK4B (Figure 1B).

### Production of Recombinant CpCDPK Proteins in *Escherichia coli*

**Figure 2:**
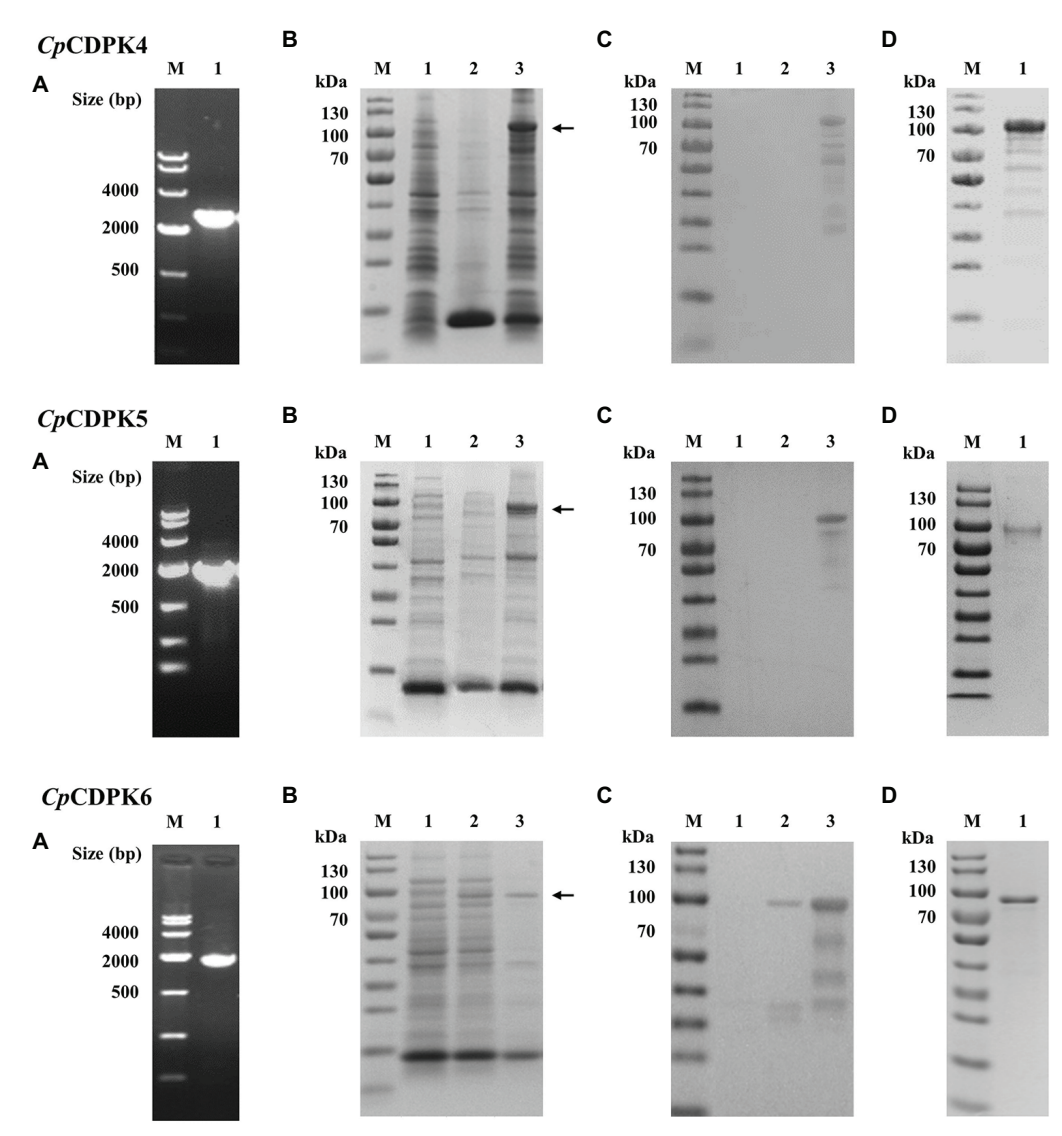
Production and purification of recombinant CpCDPK4, CpCDPK5, and CpCDPK6 proteins of *C. parvum*. **(A)** PCR amplification of the target genes of *C. parvum*. Lane M: 1,000 bp molecular markers; Lane 1: PCR product of the target gene. **(B)** Expression of recombinant CpCDPK proteins in *Escherichia coli* BL21 (DE3) as revealed by sodium dodecyl sulfate-polyacrylamide gel electrophoresis (SDS-PAGE). Lane M: protein molecular weight markers; Lane 1: lysate from recombinant bacteria without isopropylthio-β-galactoside (IPTG) induction; Lane 2: supernatant of lysate from IPTG-induced recombinant bacteria; and Lane 3: pellet of lysate from IPTG-induced recombinant bacteria, with the expected product being indicated by the black arrow. **(C)** Western blot analysis of the recombinant proteins. Lane M: protein molecular weight markers; Lane 1: lysate from recombinant bacteria without IPTG induction; Lane 2: supernatant of lysate from IPTG-induced recombinant bacteria; and Lane 3: pellet of lysate from IPTG-induced recombinant bacteria. **(D)** Purification of recombinant proteins. Lane M: protein molecular weight markers; Lane 1: purified recombinant protein from Ni-NTA affinity chromatography and SDS-PAGE extraction.

The three full-length *Cp*CDPK genes were amplified from genomic DNA of *C. parvum* (Figure 2A) and cloned into the pET-28a-c(+) vector. All proteins were expressed as the expected sizes of ~98, 85, and 76 kDa for *Cp*CDPK4, CpCDPK5, and *Cp*CDPK6, respectively (Figure 2B). The identity of the recombinant proteins was confirmed using Western blot analysis with anti-His tag (Figure 2C) and MALDI-TOF/MS analysis, which obtained peptide sequences of the study *Cp*CDPKs (data not shown). The recombinant CpCDPKs were purified using Ni-NTA beads and gel extraction (Figure 2D).

### Expression of CpCDPK Genes in *in vitro* Developmental Stages

**Figure 3:**
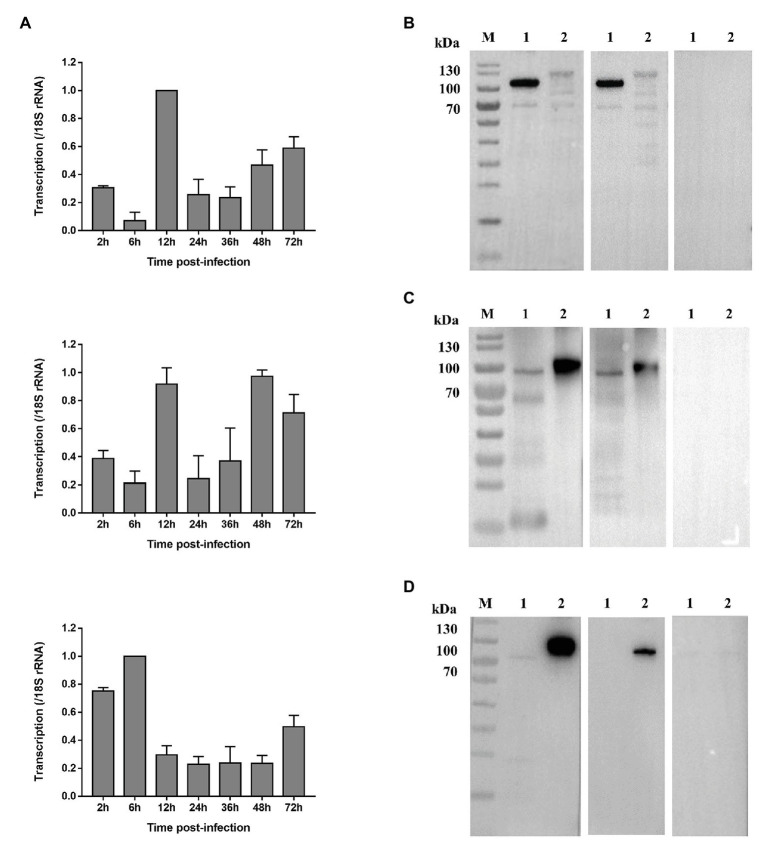
Expression of CpCDPK4, CpCDPK5, and CpCDPK6 genes in developmental stages and crude sporozoite proteins of *C. parvum*. **(A)** Expression levels of the CpCDPK4 gene (top panel), *Cp*CDPK5 gene (middle panel), and CpCDPK6 gene (bottom panel) at various time points of *C. parvum* culture. The gene expression was assessed using qPCR, with data from the *Cryptosporidium* 18S rRNA gene being used as an internal control for data normalization. Data presented are means ± SD from three independent assays. **(B)** Western blot analysis of native CpCDPK4 in *C. parvum* sporozoites with polyclonal antibodies (left panel), post immune sera (middle panel), and pre-immune sera (right panel). Lane M: protein molecular weight markers; Lane 1: purified recombinant CpCDPK4 protein; and Lane 2: crude protein extracted from sporozoites. **(C)** Western blot analysis of native CpCDPK5 in *C. parvum* sporozoites with polyclonal antibodies (left panel), post immune sera (middle panel), and pre-immune sera (right panel). Lane M: protein molecular weight markers; Lane 1: purified recombinant CpCDPK5 protein; and Lane 2: crude protein extracted from sporozoites. **(D)** Western blot analysis of native CpCDPK6 in *C. parvum* sporozoites with polyclonal antibodies (left panel), post immune sera (middle panel), and pre-immune sera (right panel). Lane M: protein molecular weight markers; Lane 1: crude protein extracted from sporozoites; and Lane 2: purified recombinant CpCDPK6 protein.

The expression levels of the CpCDPK genes in intracellular developmental stages of *C. parvum* were assessed by using qRT-PCR. For CpCDPK4, the expression level was low at early infection. At 12 h, CpCDPK4 expression increased drastically but remained low at 24 and 36 h. Afterward, CpCDPK4 expression increased gradually from 48 to 72 h (Figure 3A, top panel). For CpCDPK5, the expression level was high at 12 and 48 h, while lower expression was observed at other time points (Figure 3A, middle panel). As for CpCDPK6, the expression level was high at 2 h, peaked at 6 h, and maintained at low levels from 12 to 48 h (Figure 3A, bottom panel).

### Expression of Native CpCDPK Proteins in *in vitro* Developmental Stages

The recombinant *Cp*CDPK proteins were used to generate polyclonal antibodies and immune sera, which were used in the analysis of the expression of native *Cp*CDPKs in crude protein extract of sporozoites, with the pre-immune serum being used as the control (Figures 3B–D). All recombinant CpCDPK proteins were recognized at the expected sizes by corresponding polyclonal antibodies and immune sera. Native CpCDPK4 was recognized by the antibodies and immune sera at ~120 kDa with several smaller bands between 50 and 100 kDa (Figure 3B). In contrast, the native CpCDPK5 was recognized by the antibodies and immune sera at ~100 kDa (Figure 3C), while the native CpCDPK6 was only recognized by the anti-CpCDPK6 antibodies at ~80 kDa, but not by immune sera (Figure 3D). These recombinant and native CpCDPKs were not recognized by the pre-immune serum.

**Figure 4:**
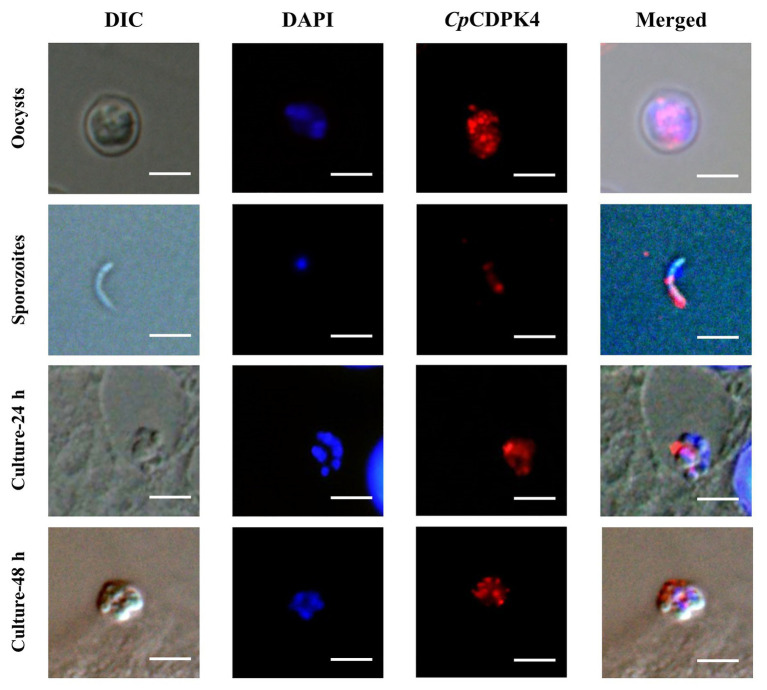
Localization of CpCDPK4 protein in lifecycle stages of *C. parvum*. Expression of CpCDPK4 in oocysts **(first panel)**, sporozoites **(second panel)**, and intracellular developmental stages in Human colon adenocarcinoma cell (HCT-8 cell) cultures at 24 h **(third panel)** and 48 h **(fourth panel)**. The images were taken under differential interference contrast (DIC), with nuclei being counter stained with 4',6-diamidino-2-phenylindole (DAPI), with parasites stained by immunofluorescence with Alexa 594-labeled CpCDPK4 (CpCDPK4), and with superimposition of the three images (Merged). Bars = 5 μm.

**Figure 5:**
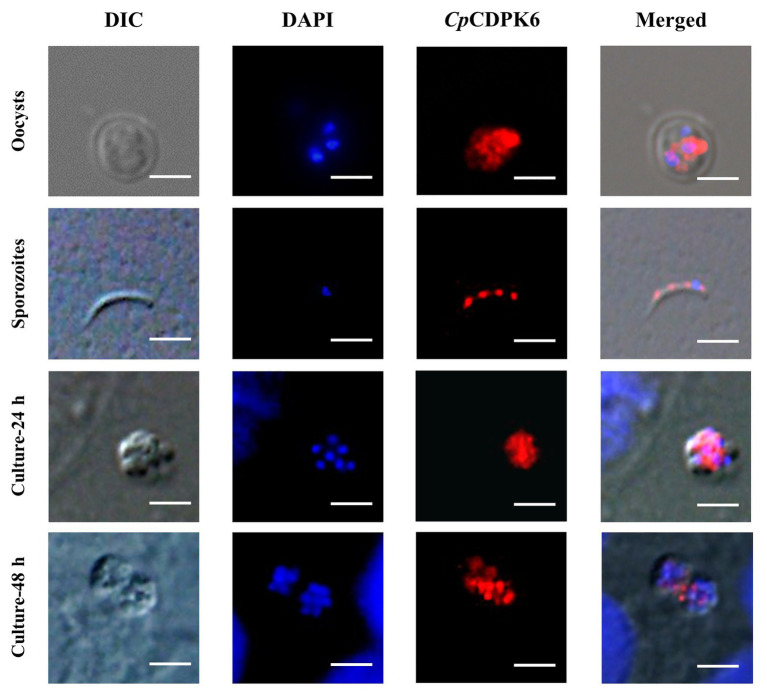
Localization of CpCDPK5 protein in lifecycle stages of *C. parvum*. Expression of CpCDPK6 in oocysts **(first panel)**, sporozoites **(second panel)**, and intracellular developmental stages in HCT-8 cell cultures at 24 h **(third panel)** and 48 h **(fourth panel)**. The images were taken under DIC, with nuclei being counter-stained with DAPI, with parasites stained by immunofluorescence with Alexa 594-labeled CpCDPK6 (CpCDPK6), and with superimposition of the three images (Merged). Bars = 5 μm.

**Figure 6:**
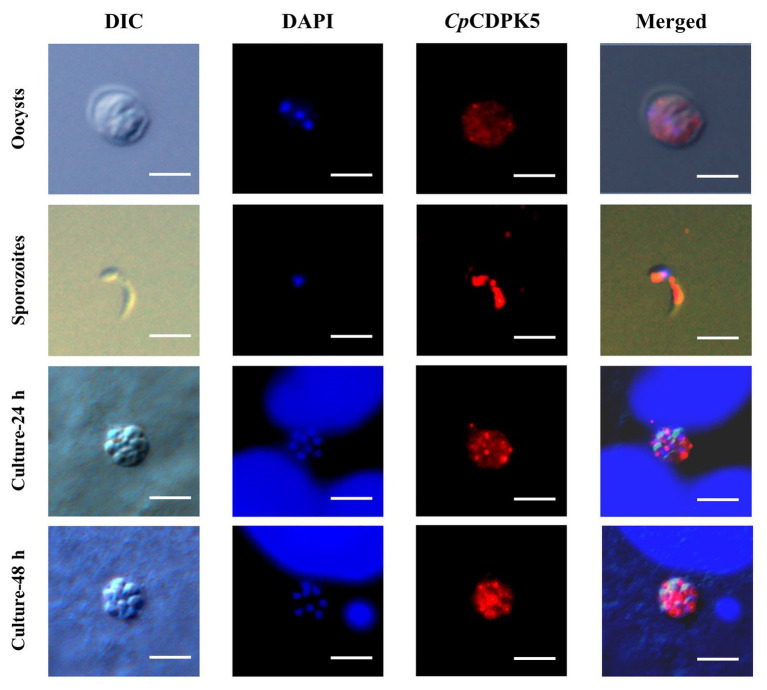
Localization of CpCDPK6 protein in lifecycle stages of *C. parvum*. Expression of CpCDPK5 in oocysts **(first panel)**, sporozoites **(second panel)**, and intracellular developmental stages in HCT-8 cell cultures at 24 h **(third panel)** and 48 h **(fourth panel)**. The images were taken under DIC, with nuclei being counter-stained with DAPI, with parasites stained by immunofluorescence with Alexa 594-labeled CpCDPK5 (CpCDPK5), and with superimposition of the three images (Merged). Bars = 5 μm.

In the examination of CpCDPK expression in developmental stages using immunofluorescence microscopy, the anti-CpCDPK4 and anti-CpCDPK6 antibodies reacted with the middle part of the oocysts strongly (Figures 4, 5, first panel), while the anti-CpCDPK5 antibodies reacted with the entire oocysts (Figure 6, first panel). In sporozoites, anti-CpCDPK4 antibodies reacted mostly with the anterior and mid-anterior regions (Figure 4, second panel), while anti-CpCDPK5 antibodies reacted with the entire sporozoites (Figure 6, second panel). In contrast, CpCDPK6 had shown spotty distribution on the sporozoites (Figure 5, second panel). In 24 and 48-h *C. parvum* cultures, the expression of CpCDPK4 was not strong and was located at only part of the merozoites opposite to the nucleus (Figure 4, third and fourth and bottom panel), while the anti-CpCDPK5 and anti-CpCDPK6 antibodies appeared to have reacted with the entire merozoites (Figures 5, 6, third and fourth panel). All antibodies against CpCDPK proteins did not recognize the parasitophorous vacuole.

### Neutralization of *Cryptosporidium parvum* Invasion by Anti-CpCDPK Antibodies

**Figure 7:**
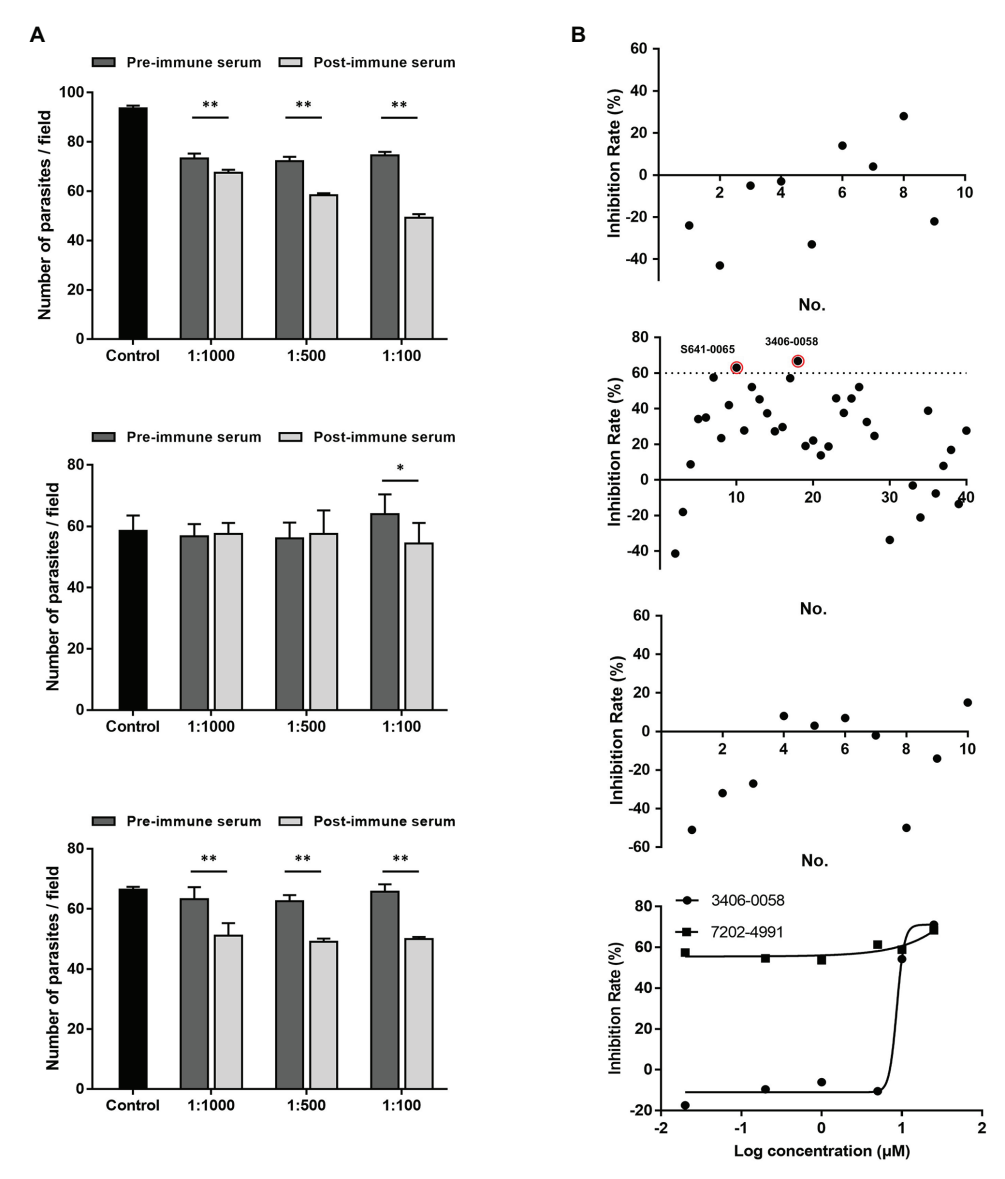
Neutralization efficiency of *C. parvum* invasion by post-immune serum against CpCDPKs and inhibitory efficacy on *C. parvum* development by candidate compounds from CpCDPK docking. **(A)** Neutralization efficiency of *C. parvum* invasion by post-immune sera against CpCDPK4 (top panel), CpCDPK5 (middle panel), and CpCDPK6 (bottom panel). The neutralization efficiency was measured in HCT-8 cell culture. Data from cultures treated with the pre-immune serum were used for comparison. Data presented are means ± SD from three independent assays. ^*^p < 0.05; ^**^p < 0.01. **(B)** Efficacy of candidate compounds from molecular dockings of CpCDPK4 (first panel), CpCDPK5 (second panel), and CpCDPK6 (third panel) at 10 μM (black dots). Compounds 3406-0058 and 7202-4991 were further assessed in dose-response experiments (fourth panel). Data presented are means from three biological replicates. Compounds with high efficacy (>60% inhibition) were highlighted with red circles.

Compared with the control cultures treated with pre-immune sera, significant reductions in *C. parvum* loads were observed in the cultures treated with the immune sera against CpCDPK4 (Figure 7A, top panel). The inhibition rates were 7.8% (73.2 ± 1.7 and 67.5 ± 1.2 per 200 × field for pre‐ and post-immune sera, respectively; *t*_(2)_ = 11.270, *p* = 0.0078) at 1:1,000 dilution, 19.2% (72.3 ± 1.4 and 58.4 ± 0.8 per 200 × field for pre‐ and post-immune sera, respectively; *t*_(2)_ = 10.924, *p* = 0.0083) at 1:500 dilution, and 34% (74.6 ± 1.1 and 49.3 ± 1.5 per 200 × field for pre‐ and post-immune sera, respectively; *t*_(2)_ = 59.705, *p* = 0.0003) at 1:100 dilution. The parasite load in cultures with no addition of any serum was 93.62 ± 1.1 per 200 × field. Similarly, the immune sera against CpCDPK6 also exhibited a significant inhibitory effect on *C. parvum* growth (Figure 7A, bottom panel). The inhibition rates were 19.2% (63.2 ± 3.3 and 51.0 ± 4.2 per 200 × field for pre‐ and post-immune sera, respectively; *t*_(2)_ = 19.567, *p* = 0.0026) at 1:1,000 dilution, 21.6% (62.5 ± 1.7 and 49.0 ± 1.1 per 200 × field for pre‐ and post-immune sera, respectively; *t*_(2)_ = 12.328, *p* = 0.0065) at 1:500 dilution, and 24% (65.7 ± 2.0 and 50.0 ± 0.7 per 200 × field for pre‐ and post-immune sera, respectively; *t*_(2)_ = 11.509, *p* = 0.0075) at 1:100 dilution. The parasite load in cultures with no addition of serum was 66.3 ± 1.0 per 200 × field. In contrast, the highest inhibition rate by the immune sera against CpCDPK5 was 14.9% (64.0 ± 6.4 and 54.4 ± 6.7 per 200 × field for pre‐ and post-immune sera, respectively; *t*_(2)_ = 7.953, *p* = 0.0155) at 1:100 dilution. The parasite load in cultures with no addition of serum was 58.6 ± 5.0 per 200 × field (Figure 7A).

### Anti-cryptosporidial Effects of Candidate Compounds from Molecular Docking of CpCDPKs

Based on the results of molecular docking, 9 compounds were selected as the candidate inhibitors for CpCDPK4, 40 compounds for CpCDPK5, and 10 compounds for CpCDPK6. These compounds were evaluated for inhibitory effects on *C. parvum* development (including both invasion and growth) at the concentration of 10 μM using a qPCR-based quantitation of parasite load in HCT-8 cell cultures. For CpCDPK4, the mean inhibition rates of these compounds were compared with the DMSO-treated controls ranging from −42.7 to 27.6%, and none of the compounds showed significant effects using a cutoff value of 60% (Figure 7B, first panel). Similar effects were observed on cultures treated with compounds for CpCDPK6, with the mean inhibition rates ranging from −50.7 to 8.1% (Figure 7B, third panel). Among the 40 compounds for CpCDPK5, two compounds (3406-0058 and 7202-4991) showed some effects on *C. parvum* development (Figure 7B, second panel).

The potency of these two compounds was further assessed in dose-response experiments. Between the two, compound 3406-0058 showed a rapid decay of effects at lower concentrations while compound 7202-4991 showed consistent effects on *C. parvum*, making the calculation of EC_50_ values for both compounds difficult (Figure 7B, fourth panel).

## Discussion

Results of the study have shown some potential involvement of CpCDPK4 and CpCDPK6 in the invasion and intracellular development of *C. parvum*, while CpCDPK5 may have different functions. In apicomplexan protozoa, CDPKs are considered attractive drug targets for cryptosporidiosis (Hui et al., 2015). However, the functions of most CDPKs were not clear. Before this, studies on CpCDPKs have focused exclusively on CpCDPK1 and CpCDPK3. In the present study, we have evaluated for the first time the functions of CpCDPK4, CpCDPK5, and CpCDPK6. Our results suggest that these CpCDPK proteins could play different roles in the lifecycle of *C. parvum*.

Data generated in the study indicate that CpCDPK4 may participate in invasion or early intracellular development of *C. parvum*. *Cryptosporidium parvum* genes are usually expressed in a stage-specific manner (Etzold et al., 2014; Lippuner et al., 2018). At the RNA level, the expression level of CpCDPK4 gene peaked at 12 h post-infection, coinciding with the emergence of immature meront (12 h; Hijjawi et al., 2001; Mauzy et al., 2012). At the protein level, CpCDPK4 is located mostly at the anterior and mid-anterior regions in sporozoites, where most invasion-associated proteins were located in (Singh et al., 2015). In *in vitro* neutralization of *C. parvum* invasion, significant reductions in *C. parvum* loads were observed in the cultures treated with the immune sera against CpCDPK4. Thus, CpCDPK4 is more likely to play some roles in the early development of *C. parvum*. Phylogenetic analysis showed that the ortholog of CpCDPK4 in *T. gondii* is TgCDPK4B, while there is no ortholog in *P. falciparum* (the PfCDPK4 is phylogenetically related to CpCDPK1 and TgCDPK1). So far, there is only one report on TgCDPK4B, which is a typical CDPK with a kinase domain and EF-hands (Long et al., 2016), constitutively expressed at low levels during the cell cycle of *T. gondii* (Long et al., 2016), indicating that TgCDPK4B might have functions different from CpCDPK4.

Several lines of evidence suggest that CpCDPK5 is probably not crucial in *C. parvum* invasion. At the RNA level, the expression of the CpCDPK5 gene peaked at 12 and 48 h, coinciding with the emergence of immature meront (mostly 12 h) and merozoite reinfection (mostly 48 h) in *C. parvum* (Hijjawi et al., 2001; Mauzy et al., 2012). At the protein level, anti-CpCDPK5 antibodies reacted with the entire surface of sporozoites. In neutralization assays, anti-CpCDPK5 polyclonal antibodies showed only limited effect at 1:100 dilution and failed to block the invasion of host cells by *C. parvum* at higher dilutions. These results are consistent with the late peak expression of the CpCDPK5 gene in the lifecycle of the pathogen. They also agree with observations on CpCDPK3, which was shown to participate only in the intracellular growth of *C. parvum* in a previous study (Zhang et al., 2020). The role of CpCDPK5 in *C. parvum* appears to be similar to the function of TgCDPK5 and PfCDPK5, which are orthologs of CpCDPK5. PfCDPK5 was reported to regulate *P. falciparum* egress from erythrocytes and play an essential role during the blood stage of malaria replication (Dvorin et al., 2010; Lasonder et al., 2015; Rout and Mahapatra, 2019; Ghartey-Kwansah et al., 2020). It is localized within micronemes and is required for their discharge (Absalon et al., 2018). Recently, PfCDPK5 was identified as one of the proteins related to the survival of *P. falciparum* and thus is considered a target for drug development (Zhang et al., 2014a). To date, there are only three reports on TgCDPK5. Zhang associates evaluated the immune protection induced by vector pVAX-TgCDPK5 in mice. The results demonstrated that pVAX-TgCDPK5 can elicit strong humoral and cellular immune responses and slightly prolonged the survival of immunized mice when challenged with *T. gondii* (Zhang et al., 2014a). In a study of the expression of TgCDPKs, Wang et al. (2015) associates observed that the expression of TgCDPK5 changed in response to microneme proteins, suggesting the CDPK might be involved in host cell invasion, egress, and motility. Another study showed that there was no significant difference between TgCDPK5 knockout strains and the wild-type strain in virulence, which indicated that TgCDPK5 might have other functions in *T. gondii* (Wang et al., 2016). The sequence similarity among CDPK5 proteins of apicomplexans suggests that CpCDPK5 might have functions similar to PfCDPK5 and TgCDPK5.

The role of CpCDPK6 could be different from that of CpCDPK4 and CpCDPK5. At the RNA level, the expression of the CpCDPK6 gene peaked during early *C. parvum* infection. At the protein level, the localization of CpCDPK6 showed a punctate distribution on sporozoites. In *in vitro* assays, immune sera against CpCDPK6 exhibited a significant inhibitory effect on *C. parvum* invasion. These lines of evidence indicate that CpCDPK6 may be involved in the invasion and intracellular development. In other apicomplexans, TgCDPK6 and PfCDPK6 shares high homology with CpCDPK6. PfCDPK6 was shown to be involved in the transition to an invasive phenotype of *P. falciparum*. Sporozoites of PfCDPK6 mutants showed enhanced migratory activities and were significantly less infective to hepatocytes. Moreover, they were deficient in cleaving the major surface protein of sporozoites (Coppi et al., 2007). TgCDPK6 mutants had a mild defect of growth, resulting in lower tissue cyst burden (Long et al., 2016). In another study, TgCDPK6 was shown to not be involved in virulence and the lytic cycle, which includes egress, invasion, and replication (Wang et al., 2016). The expression pattern of TgCDPK6 indicated that it could be involved in oocysts development (Wang et al., 2015).

To date, studies of inhibitors for CpCDPKs have focused on CpCDPK1, including pyrazolopyrimidine (PP) analogs and bumped kinase inhibitors (BKIs). CpCDPK1 is sensitive to these inhibitors due to the unique smaller gatekeeper in its ATP-binding pocket, while other CpCDPKs do not have this structure; thus, they are not susceptible to these compounds (Wernimont et al., 2010; Artz et al., 2011; Zhang et al., 2014b). In our previous study, we assessed the inhibitory effects of candidate compounds from molecular docking of CpCDPK3 on *C. parvum* development and CpCDPK3 enzyme activities and identified one effective compound (Zhang et al., 2020). In this study, we tested 9 candidate compounds for CpCDPK4, 40 candidate compounds for CpCDPK5, and 10 candidate compounds for CpCDPK6 based on the results of molecular docking. Only two compounds for CpCDPK5 (3406-0058 and 7202-4991) showed some effects on *C. parvum* development. However, the effect of the two compounds on CpCDPK5 could not be assessed due to the lack of enzyme activities of the recombinant CpCDPK5 protein generated in this study. Recent studies on in *Plasmodium* spp. have shown some overlapping functions of various CDPKs, which could have reduced the inhibitory effects of these potential CDPK inhibitors on *in vitro* parasite growth (Blackman et al., 2020; Ghartey-Kwansah et al., 2020).

## Conclusion

Our findings suggest that CpCDPKs may play different roles in the *Cryptosporidium* lifecycle. Although we have obtained some preliminary evidence to support the suggestion, additional studies using more advanced tools such as genetic manipulation of the pathogen by CRISPR/Cas9 are needed. Moreover, it is necessary to conduct quantitative assessment of the expression of CpCDPKs among lifecycle stages and to obtain active recombinant proteins for functional studies. These studies are likely leading to improve the understanding of functions of CpCDPKs and the development of new drug targets.

## Data Availability Statement

The original contributions presented in the study are included in the article/supplementary material and further inquiries can be directed to the corresponding authors.

## Author Contributions

YF and LX designed the study. QZ, QS, JS, and RX performed the experiments and statistical analysis. YL and ZZ performed the molecular docking work. NL and YG provided technical assistance. QZ, YF, and LX developed the manuscript. All authors contributed to the article and approved the submitted version.

### Conflict of Interest

The authors declare that the research was conducted in the absence of any commercial or financial relationships that could be construed as a potential conflict of interest.

## Footnotes

^1^http://cryptodb.org

^2^https://www.ebi.ac.uk/Tools/hmmer

^3^https://imagej.nih.gov/ij/
